# Using hospital discharge data to identify incident pregnancy-associated cancers: a validation study

**DOI:** 10.1186/1471-2393-13-37

**Published:** 2013-02-11

**Authors:** Yuen Yi Cathy Lee, Christine L Roberts, Jane Young, Timothy Dobbins

**Affiliations:** 1 Clinical and Population Perinatal Health Research, Kolling Institute of Medical Research, University of Sydney, New South Wales, Australia; 2 Cancer Epidemiology and Services Research Group, University of Sydney, New South Wales, Australia

**Keywords:** Cancer, Pregnancy, Incidence, Sensitivity, Positive predictive value, Validation

## Abstract

**Background:**

Pregnancy-associated cancer is associated with maternal morbidities and adverse pregnancy outcomes, and is reported to be increasing. Hospital discharge data have the potential to provide timely information on cancer incidence, which is central to evaluation and improvement of clinical care for women. This study aimed to assess the validity of hospital data for identifying incident pregnancy-associated cancers compared with incident cancers from an Australian population-based statutory cancer registry.

**Methods:**

Birth data from 2001–2008, comprised 470,277 women with 679,736 maternities, were linked to cancer registry and hospitalisation records to identify newly diagnosed cancers during pregnancy or within 12 months of delivery. Two hospital-identified cancer groups were examined; “index cancer hospitalisation” – first cancer admission per woman per pregnancy and “all cancer hospitalisations” –the total number of hospitalisations with a cancer diagnosis and women could have multiple hospitalisations during pregnancy. The latter replicates a scenario where identification of individuals is not possible and hospitalisations are used as the unit of analysis.

**Results:**

The incidence of pregnancy-associated cancer (according to cancer registry) was 145.4/100,000 maternities. Incidence of cancer was substantially over-estimated when using hospitalisations as the unit of analysis (incidence rate ratio, IRR 1.7) and under-estimated when using the individual (IRR 0.8). Overall, the sensitivity of “index cancer hospitalisation” was 60.4%, positive predictive value (PPV) 77.7%, specificity and negative predictive value both 100%. Melanoma ascertainment was only 36.1% and breast cancer 62.9%. For other common cancers sensitivities ranged from 72.1% to 78.6% and PPVs 56.4% to 87.3%.

**Conclusion:**

Although hospital data provide another timely source of cancer identification, the validity is insufficient to obtain cancer incidence estimates for the obstetric population.

## Background

Cancers associated with pregnancy are reported to be increasing, and this has been attributed to increasing maternal age and women’s interaction with health services during pregnancy [1]. Women with cancer diagnosed during pregnancy or within 12 months of delivery (referred to as ‘pregnancy-associated cancer’) are at increased risk of maternal morbidities and adverse pregnancy outcomes [1,2]. Current information on incidence of cancer associated with pregnancy is central to evaluation and improvement of clinical care for women.

Population-based statutory cancer registries are a reliable source for identifying incident cancers in populations [3-5]. However, the extensive quality assurance processes implemented by cancer registries can delay the timely availability of cancer data [6]. Consequently, there is increasing interest in the use of routinely collected and easily accessible administrative data, such as hospital discharge data, for identifying incident cancers and assessing health service utilisation and quality of care for cancer patients [3-5,7]. One disadvantage of using databases of hospitalisation records is that individuals with multiple hospitalisations cannot always be identified. For example, several obstetric research studies in the United States (US) to date have relied solely on hospitalisation records, which means 100 records may come from 100 individuals or 10 individuals each admitted 10 times [8-10]. Further complications arise in identifying pregnancy-associated cancers from hospital data due to the inability to determine the duration of pregnancy in weeks of gestation, resulting in an imprecise pregnancy exposure period [8].

The quality of estimates of hospital-ascertained cancer incidence relies on complete and accurate ascertainment of recorded diagnoses. However, the validity of hospital data for identifying incident cancers associated with pregnancy has yet to be established. Several US studies have validated Medicaid or Medicare claims data (inpatient, outpatient and physician claims) for identification of incident cancers with cancer registries as the “gold standard”, with sensitivities ranging from 68% to 97% and positive predictive values ranging from 83% to 96% [11-16]. There have also been limited efforts to link private insurer claims to cancer registries, with a focus on cancer treatment information [17]. The generalisability of such findings to specific populations (e.g., obstetric populations) remains questionable, given that the majority of published studies are among elderly populations. Therefore, the aim of this study was to determine the validity of hospital diagnoses for identification of incident pregnancy-associated cancers, both overall and by cancer type, compared with incident diagnoses from a population-based statutory cancer registry.

## Methods

### Data sources and study population

The study population comprised 470,277 women who gave birth in New South Wales (NSW) in the period 2001 to 2008, which corresponded to 679,736 maternities. NSW is the most populous state of Australia with a resident population of approximately 7 million people. Approximately one-third of all Australian births occur in NSW public or private hospitals.

Data were obtained from three linked NSW population databases: the Perinatal Data Collection (PDC), Admitted Patient Data Collection (APDC) and Central Cancer Registry (CCR). The PDC is a statutory surveillance system that includes births of at least 20 weeks gestation or at least 400 grams birth weight. The APDC (referred to as “hospital data”) is a census of all inpatient hospitalisations to NSW public and private hospitals. The CCR (referred to as “registry data”) is a statutory case-based registry of all newly diagnosed cancers in NSW since 1972 with the exception of non-melanoma skin cancers. Cancer diagnoses in the CCR are coded according to the 3^rd^ edition of the International Classification of Diseases for Oncology [18]. The registry data are validated by a rigorous procedure to ensure the completeness and quality of data are high. Over 90% of cancers are verified by pathology and are confirmed as the primary cancer diagnosis. Less than 1% of cancers are ascertained from the death certificate [19]. Based on treatment categories, cancers were categorised into 13 clinical groupings [19]. For validation purposes, we used the registry data as the ‘gold standard’ of identification of incident cancers associated with pregnancy.

Record linkage of the three databases was carried out by the NSW Centre for Health Record Linkage using probabilistic record linkage methods [20,21]. This involves a process of blocking and matching combinations of selected variables with identifying information such as name, date of birth, address and hospital. Each match was assigned with a probability weight [22]. The probabilistic record linkage for this study is highly reliable with less than three in 1,000 false positive links and less than five in 1,000 missed links [20]. The researchers were provided anonymised data. The study was approved by the NSW population and Health Services Research Ethics Committee.

The PDC birth records were used to identify women who gave birth from 2001 to 2008 and to obtain duration of pregnancy in completed weeks of gestation. The birth records were then linked to the cancer registry and hospitalisation records to identify newly diagnosed cancers during pregnancy or within 12 months of delivery [1,2]. For identification of pregnancy-associated cancers from hospital data we searched up to 22 diagnosis fields associated with each pregnancy and postpartum hospital admission for cancer diagnoses. The diagnosis fields were coded according to the ICD-10-AM coded (10^th^ revision of the International Classification of Disease, Australian Modification ) with cancer diagnoses indicated by codes of C00–C96 [23]. As our aim was to identify incident cancers, any hospitalisation record coded as secondary, in-situ or benign cancer was excluded from the analysis. Hospital-identified pregnancy-associated cancers were examined based on two groups: i) “index cancer hospitalisation”, identified by taking the record with the earliest admission date for each cancer type. This replicates a scenario where individuals can be identified in the hospital data and the first record is considered to be an incident cancer; ii) ‘all cancer hospitalisations’, refers to the total number of hospitalisations with a cancer diagnosis and women could have multiple hospitalisations during pregnancy. This replicates a scenario where identification of individuals is not possible and hospitalisations (records) are used as the unit of analysis.

### Statistical analysis

The incidence estimated from ‘all cancer hospitalisations’ and the ‘index cancer hospitalisation’ were compared with the incidence estimated from registry data (both overall and by cancer type). The ratios of the hospital cancer incidence to the registry cancer incidence (denoted incidence rate ratio, IRR) were calculated. To assess the validity of the hospital identification of incident pregnancy-associated cancers only ‘index cancer hospitalisation’ data were compared with registry data. The following reporting characteristics with 95% exact binomial confidence interval (95%CI) were computed: sensitivity, specificity, positive predictive value (PPV) and negative predictive value (NPV). Sensitivity (sometimes referred to as true positive fraction) is the proportion of maternities with an associated cancer, as ascertained by the registry gold standard, that were also identified in the hospital data, thus measuring completeness of identification. Specificity (equivalent to one minus false positive fraction) is the proportion of maternities without an associated cancer that were also not identified in the hospital data. PPV is the proportion of maternities with an associated cancer identified in the hospital data that in fact had an associated cancer. NPV is the proportion of maternities without an associated cancer identified in the hospital data that in fact did not have an associated cancer. We compared admission date and registry date among true-positive pregnancy-associated cancers to quantify the discrepancy between hospital and registry data regarding the timing of cancer incidence. In addition, we examined the false-positive and false-negative pregnancy-associated cancers in the hospital data (compared with registry data) to determine if some of these were prevalent cancers or had an inconsistent cancer type. To differentiate truly incident from false-positive cancers, we compared the hospital-identified cancers with registry data from 1994 onwards and regarded those previously notified cancers of the same type as prevalent cancers. Analysis was carried out in SAS, Version 9.2 (SAS Institute, Cary NC, USA) [24].

## Results

### Incidence of pregnancy-associated cancer from registry and hospital data

Between 2001 and 2008, a total of 988 pregnancy-associated cancers were identified from the registry data, corresponding to an overall crude incidence of 145.4 per 100,000 maternities (Table 1). The resultant incidence estimates of hospital-identified pregnancy-associated cancer were notably higher among “all cancer hospitalisations” (252.7 per 100,000 maternities, IRR = 1.7) and lower among the “index cancer hospitalisations” (113.3 per 100,000 maternities, IRR = 0.8) (Table 1). Breast and lymphohaematopoeitic cancers resulted in large number of hospitalisations. The most common cancer in the registry data was melanoma with an incidence of 47.4 per 100,000 maternities, which was two times higher than that reported in the “index cancer hospitalisation” (20.9 per 100,000 maternities, IRR = 0.4). The next most common cancers were breast (32.1 per 100,000 maternities in registry data) and thyroid and other endocrine cancers (19.6 per 100,000 maternities), and these rates were also comparatively higher than that reported in the “index cancer hospitalisation” (22.4 and 17.5 per 100,000 maternities, and IRR = 0.8 and 0.9 respectively) (Table 1).

**Table 1:** Pregnancy-associated cancer rates in the registry and hospital discharge datasets

**Clinical group of cancer**	**Incidence of pregnancy-associated cancer by data source**										
	**Cancer Registry**^**a**^			**All hospitalisations**^**b**^				**Index hospitalisation**^**b**^			
	***N***	***%***	***Rate***	***N***	***%***	***Rate***	***IRR***^***c***^	***N***	***%***	***Rate***	***IRR***^***c***^
Melanoma	322	32.6	47.4	168	9.8	24.7	0.5*	142	18.4	20.9	0.4*
Breast	218	22.0	32.1	491	28.6	72.2	2.3*	152	19.7	22.4	0.7*
Thyroid and other endocrine	133	13.5	19.6	198	11.5	29.1	1.5*	119	15.5	17.5	0.9
Gynaecological	94	9.5	13.8	161	9.4	23.7	1.7*	81	10.5	11.9	0.9
Lymphohaematopoeitic	87	8.8	12.8	340	19.8	50.0	3.9*	110	14.3	16.2	1.3
Colorectal	35	3.5	5.1	44	2.6	6.5	1.3	22	2.9	3.2	0.6
Neurological	22	2.2	3.2	57	3.3	8.4	2.6*	24	3.1	3.5	1.1
Bone and other connective tissue	19	1.9	2.8	64	3.7	9.4	3.4*	25	3.2	3.7	1.3
Head and Neck	14	1.4	2.1	13	0.8	1.9	0.9	11	1.4	1.6	0.8
Upper Gastrointestinal	18	1.8	2.6	124	7.2	18.2	6.9*	52	6.8	7.7	3.0*
Respiratory	7	0.7	1.0	17	1.0	2.5	2.4	9	1.2	1.3	1.3
Ill-defined and unknown primary sites	11	1.1	1.6	22	1.3	3.2	2.0	12	1.6	1.8	1.1
Urogenital	8	0.8	1.2	19	1.1	2.8	2.4	11	1.4	1.6	1.3
Any	988	100	145.0	1,718	100	252.7	1.7*	770	100	113.3	0.8*

Rates are expressed per 100,000 maternities.

^a^Two women had multiple cancer notifications of different type in a pregnancy.

^b^Twenty-three women had multiple cancer hospitalisations of different type in a pregnancy.

^c^ Incidence rate ratio^:^ The ratio of hospital cancer incidence to registry cancer incidence.

(* denotes statistical significance at *P* < 0.05).

### Reporting characteristics of pregnancy-associated cancer

Overall, the sensitivity of hospital record identification of cancer was 60.4% and PPV was 77.7% (Table 2), and excluding melanoma were 72.5% and 77.2% respectively. The sensitivities for the majority of pregnancy-associated cancers were over 70.0%, except for melanoma (36.1%), breast (62.9%), colorectal (64.3%) and head and neck cancers (69.2%) (Table 2). Respiratory and urogenital cancers had 100% sensitivities, although their sample sizes were small. There was some variation in positive predictive values by cancer type. Breast, thyroid and other endocrine, gynaecological, neurological and head and neck cancers had PPVs ranging from 81.8% to 87.3%. For the remaining cancers, PPV ranged from 56.4% for lymphohaematopoeitic cancer to 79.9% for melanoma (Table 2). Due to the rarity of pregnancy-associated cancer, the specificities and negative predictive values ranged from 99.9% to 100% (data not shown).

**Table 2:** Reporting characteristics of incident pregnancy-associated cancer identified in hospital data compared with cancer registry data

**Clinical group of cancer**	**Reporting characteristics of pregnancy-associated cancer**						
	**TP (*N*)**	**FP (*N*)**	**FN (*N*)**	**Sn (*%*)**	**95% CI (*%*)**	**PPV (*%*)**	**95% CI (*%*)**
Melanoma	115	29	204	36.1	[30.8,41.6]	79.9	[72.4,86.1]
Breast	134	20	79	62.9	[56.0,69.4]	87.0	[80.7,91.9]
Thyroid and other endocrine	103	15	28	78.6	[70.6,85.3]	87.3	[79.9,92.7]
Gynaecological	70	15	20	77.8	[67.8,85.9]	82.4	[72.6,89.8]
Lymphohaematopoeitic	62	48	24	72.1	[61.4,81.2]	56.4	[46.6,65.8]
Colorectal	18	11	10	64.3	[44.1,81.4]	62.1	[42.3,79.3]
Neurological	20	4	2	90.9	[70.8,98.9]	83.3	[62.6,95.3]
Bone and other connective tissue	16	7	2	88.9	[65.3,98.6]	69.6	[47.1,86.8]
Head and Neck	9	2	4	69.2	[38.6,90.9]	81.8	[48.2,97.7]
Upper Gastrointestinal	15	5	3	83.3	[58.6,96.4]	75.0	[50.9,91.3]
Respiratory	6	3	0	100	[54.1,100 ]	66.7	[29.9,92.5]
Ill-defined and unknown primary sites	4	4	4	50.0	[15.7,84.3]	50.0	[15.7,84.3]
Urogenital	8	3	0	100	[63.1,100 ]	72.7	[39.0,94.0]
Any	580	166	380	60.4	[57.2,63.5]	77.7	[74.6,80.7]

*TP*: True positive; *FP*: False positive; *FN*: False negative; *Sn*: Sensitivity; *PPV*: Positive predictive value; *CI*: Confidence interval.

Note: The specificities and negative predictive values of all cancers ranged from 99.9% to 100%.

Of the 580 true-positive pregnancy-associated cancers, 66% had a corresponding cancer-associated admission date in the same month as the registry, 28% had an admission date 1–2 months prior to registration, 5% had 3–5 months prior to registration and only 1% had ≥ 6 months prior to registration.

### Investigation of false-positive and false-negative pregnancy-associated cancers

Compared with registry data, there were 166 false-positive and 380 false-negative pregnancy-associated cancers among “index cancer hospitalisations” (Table 2). False-positive cancers were predominately lymphohaematopoeitic cancer (56.4%, *n* = 48) (Table 2). Of the 166 false positives, registry data indicated that 86 (51.8%) were prevalent cancers and 22 (13.3%) were inaccurate/inconsistent identification of the cancer type. The cancers that were most frequently misclassified in the hospital data were colorectal cancer (*n =* 7, misclassified as upper gastrointestinal cancer), followed by melanoma (*n* = 3, misclassified as breast or bone and other connective tissue cancers) and gynecological cancer (*n* = 4, misclassified as upper gastrointestinal cancer). False-positive prevalent cancers were primarily lymphohaematopoeitic (38 out of 86, 44.2%), melanoma (13, 15.1%), breast (14, 16.3%) and thyroid and other endocrine cancers (10, 11.6%). When examining the false-positive cancers by type and year of admission, there was no systematic variation in the distributions of false positives.

False-negative cancers were predominantly melanoma (53.7%, *n* = 204) (Table 2). Of the 380 false negatives, 60.3% were in-situ/localised melanoma and 24.7% were regionalised/distant cancers of other types.

## Discussion

This record linkage-based validation study determined the validity of ICD-10-AM hospital diagnoses in estimating the incidence of pregnancy-associated cancer. We have demonstrated that using hospitalisations as the unit of analysis, rather than the individual, substantially over-estimated the incidence of pregnancy-associated cancer. In contrast, among individual-level hospital data the overall incidence was under-estimated. Specifically, the incidence of melanoma and breast cancer was under-estimated by approximately half. The overall low sensitivity was due to the predominance of melanoma, and that melanoma was the major contributor to the false negatives (frequently missed in the hospital data). Other common cancers (breast, thyroid, gynaecological and lymphohaematopoeitic cancers) achieved only moderate levels of validity (ascertainment 72.1–78.6% and PPV 56.4–87.3%).

There is no literature based on obstetric populations available for direct comparison of our reporting characteristics. Published studies have predominantly assessed the use of Medicare claims data compared with medical records or cancer registries for ascertainment of selected cancers (mainly breast cancer) with varying sensitivities and positive predictive values, depending on the definitions used, the study timeframe and identification algorithms (first diagnostic code only, all diagnostic codes or a combination of diagnostic or surgical procedure codes) [11-16]. Studies using ICD-9 coded hospital discharge data to identify incident breast, colorectal and lung cancers with cancer registries as the “gold standard” achieved better reporting characteristics than in our obstetric population [3,16,25]. For example, for breast cancer the sensitivity was 77%–85% PPV 57%–91%, colorectal cancer: sensitivity 72% PPV 60%–88% and lung cancer: sensitivity 81% PPV 59%–79%. A study comparing ICD-10 coded diagnoses in hospital discharge data with medical records as the “gold standard” reported a much greater validity (breast cancer: sensitivity 96% PPV 94%, colon cancer: sensitivity 93% PPV 96% and lung cancer: sensitivity 97% PPV 94%) than we report using ICD-10 for an obstetric population [26]. This is likely due to an older, non-specific hospitalised study population, which in general has a different pattern of hospital activity from an obstetric population. In NSW between 2001 and 2008, about 16% women with singleton pregnancy and 64% women with multifetal pregnancies were admitted to hospital at least once during 20–36 gestation weeks [27,28].

The evaluation of false-positive and false-negative pregnancy-associated cancers was insightful in considering the poorer identification of cancer from the hospital records of pregnant women. In accordance with literature, the common reasons for false positives were prevalent cancers or misclassification of cancer type [4]. We were unable to evaluate the underlying causes of false-negative cancers as information on the source of cancer diagnosis was not available. Others have speculated that the false-negative (missed) cancers may be due to cancers notified from death certificate or other non hospital-based institutions [4]. In our obstetric population the high proportion of in-situ/localised cancers suggests outpatient cancer management may be an important reason for false negatives. Furthermore, cancer may not be the reason for hospitalisation of a pregnant woman and diagnoses that are not relevant to the current admission are not required to be coded [29].

The strength of our study is the use of ICD-10 for coding hospital diagnoses. ICD-10 codes, based on surgical speciality, allow more detailed diagnoses coding than ICD-9 codes [30]. Importantly, there is a complete registration of cancers in the NSW statutory data collection, which provided the gold standard of identification of incident pregnancy-associated cancers without the need of an independent validation source. The linkage to cancer registry dating back to 1994 provided a unique opportunity to assess extensive history of cancer for identification of prevalent cancers and to mitigate their impact on the positive predictive values [4,5]. Unlike other studies which focused on specific cancers, our comparison of reporting characteristics was done overall and by cancer type, and accounted for women with multiple primary cancers in a pregnancy. Australia has the highest incidence rate of melanoma in the world [19], and the results excluding melanoma (with a higher overall reporting sensitivity) may be more generalisable to other countries. Several limitations of our study also deserve consideration. The number of pregnancy-associated cancers was somewhat under-estimated as early pregnancy loss (miscarriage or abortion) was not registered in the birth data. The hospital data represent only inpatient stays; it may be less complete in capturing cancers where outpatient diagnosis and treatment are more common, e.g., melanoma. However, the data also provide a general representation of inpatient stays regardless of age or insurance status [16], and they are more assessable than the primary health utilisation data. Finally, as the reporting characteristics are sensitive to the sample size, careful interpretation is needed for cancers with small sample sizes.

## Conclusions

The timely availability of population level data is a key factor in surveillance [31]. In 2012, NSW cancer registry data were available for linkage to the end of 2008, while hospital data were available through June 2011 [20]. Unfortunately, we do not consider the validity of pregnancy-associated cancers as determined by our hospital data of sufficient to be relied on for contemporary cancer incidence estimates.

Our study shows that the use of hospital data for identifying incident pregnancy-associated cancers achieved only moderate levels of validity. Although hospital data may provide another source of cancer identification for a cancer registry there will still need to be rigorous assurance to confirm cancers in order to obtain valid estimates of incidence.

## Competing interests

The authors declare no conflicts of interest.

## Authors’ contributions

YYL took responsibility for the integrity of data, the design of study, the accuracy of data analysis and drafting of the manuscript. CLR participated in conception and study design, analysis and interpretation of data, and drafting of the manuscript. TD and JY were involved in study design, acquisition of data and critical revision of the manuscript for important intellectual content. All authors read and approved the final manuscript.

## Pre-publication history

The pre-publication history for this paper can be accessed here:

http://www.biomedcentral.com/1471-2393/13/37/prepub
